# Determination of Vitamin E in Cereal Products and Biscuits by GC-FID

**DOI:** 10.3390/foods7010003

**Published:** 2018-01-01

**Authors:** Ioannis N. Pasias, Ioannis K. Kiriakou, Lila Papakonstantinou, Charalampos Proestos

**Affiliations:** 1 General Chemical Lab of Research and Analysis of Lamia, Timfristou 181, 35100 Lamia, Greece; iopas@chem.uoa.gr; 2 Chemical Laboratory of Lamia, Karaiskaki 85, 35100 Lamia, Greece; info@lamialab.com; 3 Artolife-Bionatural SA, ArmaThivon, 32200 Thiva, Greece; info@artolife.gr; 4 Food Chemistry Laboratory, Department of Chemistry, National and Kapodistrian University of Athens, Panepistimiopolis Zografou, 15771 Athens, Greece

**Keywords:** vitamin E, GC-FID, uncertainty, cereal products, reference daily value, health claim

## Abstract

A rapid, precise and accurate method for the determination of vitamin E (α-tocopherol) in cereal products and biscuits has been developed. The uncertainty was calculated for the first time, and the methods were performed for different cereal products and biscuits, characterized as “superfoods”. The limits of detection and quantification were calculated. The accuracy and precision were estimated using the certified reference material FAPAS T10112QC, and the determined values were in good accordance with the certified values. The health claims according to the daily reference values for vitamin E were calculated, and the results proved that the majority of the samples examined showed a percentage daily value higher than 15%.

## 1. Introduction

Vitamin E is a fat-soluble vitamin found in many foodstuffs, such as cereals, eggs, olive oils, and vegetables. Vitamin E occurs in many different forms (α-, β-, γ- and δ-tocopherols and α-, β-, γ- and δ-tocotrienols) and has many health benefits; it is mostly used for treating and preventing heart diseases [1,2]. It is a well-known antioxidant, preventing different types of cancer, such as lung and oral cancer and others. It is also believed to help patients with Alzheimer’s disease and other types of diseases related to the nervous system. According to the European Regulation 1169/2011 on the provision of food information to consumers, there is a daily reference intake for vitamin E equal to 12 mg per 100 g. As a rule, to decide whether a food has a significant amount of vitamin E, it must contain 15% of the reference dose per 100 g, or 1.8 mg per 100 g [3]. The claim suggests that “vitamin E contributes to the protection of cells from oxidative stress” and can only be used for food that is at least a source of vitamin E.

Nowadays, there is a great interest in functional foods, commonly known as “superfoods”. There is no clear definition for superfoods; it is a term used for foods that combine the nutritional health benefits and disease prevention of different foods [4]. These foods are mainly composed of cereals, fruits, beans and vegetable oils [5,6,7,8,9,10]. Vitamin E occurs in all these foodstuffs, but its content depends on the superfood composition. For this reason, it is of great importance to determine the appropriate content of the different food components in order to take the recommended daily dose of vitamin E without spoiling the taste or the flavor of the foodstuffs.

There are many methods for the determination of vitamin E. Liquid and gas chromatography methods with different detectors are the most common methods used nowadays for the determination of fat-soluble vitamins [11,12,13,14,15,16]. The need for the simultaneous determination of the majority of vitamins has led to the use of modern analytical methods with low limits of detection, such as Liquid Chromatography tandem mass spectroscopy (LC-MS/MS), Liquid chromatography mass spectrometry (LC-MS) and Gas Chromatography (GC × GC) methods [17,18,19]. All these methods require complicated preparation steps in order to decrease the interferences provided by the matrix.

In this article, a rapid, precise and accurate method for the determination of vitamin E in cereal products by Gas Chromatography coupled to Flame ionization Detection (GC-FID) is presented. The method is based on the method presented by Pyka et al. (2001) for the determination of α-tocopherol in human plasma and has now been modified and expanded for the determination of α-tocopherol in cereal products and biscuits [1]. The developed method is simple and rapid and can be applied for the determination of α-tocopherol in cereal products and biscuits with low limits of detection and high accuracy and precision. The method was applied in a vast number of new nutraceutical and functional cereal products and biscuits that can be characterized as superfoods, and possible health claims were examined.

## 2. Materials and Methods

### 2.1. Chemicals

All reagents used were of analytical grade. Vitamin E standard solution and ethanol were purchased from Sigma-Aldrich (Darmstadt, Germany). Hexane was purchased from VWR (Brooklyn, NY, USA) and dichloromethane was purchased from Lach-ner, s.r.o. (Neratovice, Czech Republic).

### 2.2. Instumentation

All experiments were performed with a Shimadzu GC 2010 PLUS, GC-FID system, using an Agilent DB-1 (30 m × 0.32 mm × 1 μm). The optimized conditions were as follows: injection volume of 2 μL in splitless mode; pulse time of 1.0 min; injector temperature of 300 °C; carrier gas: He at a constant flow of 2.0 mL/min; detector temperature of 340 °C. The initial oven temperature, 120 °C, was held for 1 min and was then programmed at 27 °C/min to 320 °C, at which it was held for 15 min.

### 2.3. Sample Preparation

Eighteen different cereal and bakery products, belonging to the class of superfoods were selected for the vitamin analysis. The ingredients of each product are presented in Table 1. The samples were milled and well homogenized. A sample amount of a well-homogenized cereal product (0.5000 g) was diluted in 1 mL of ethanol and left overnight in the dark. The solution was then mixed well with an appropriate amount of hexane and dichlomethane (90%/10% *v/v*) and centrifuged at 2500 rpm for 10 min, and the hexane/dichloromethne stage was evaporated to dryness at 40 °C. Finally the sample was dissolved in 1 mL of ethanol, and 2 μL was injected into the GC system.

### 2.4. Method Validation

Quantification was performed by different dilutions of the stock solution (from 0.5 to 250 mg/L). Every standard solution was measured in triplicate. Limit of Detection (LOD) and Limit of Quantification (LOQ) were calculated by the standard deviation of the intercept. Precision and accuracy experiments were carried out, and the relative standard deviation (%RSD) values were calculated from the multiple analysis of the certified reference material (FAPAS T10112QC) (*n* = 6) under repeatability and reproducibility conditions. The uncertainty of the method was also calculated on the basis of the Eurachem/Citac Guidelines [20].

## 3. Results and Discussion

### 3.1. The Results of Method Validation

For the determination of α-tocopherol, content quantification was performed by plotting the concentration (C) of different calibration standards (0.5–50 mg/L) versus the peak area of the analyte (PA). The equation of the calibration curve was found to be PA = (7.08 ± 0.16) × 10^4^C (mg/L) (1.5 ± 3.0) × 10^3^, *R*^2^ = 0.998. It must be noted that an internal quality control sample was measured every 10 samples in order to avoid the use of an internal standard. All samples and the standard solution were measured in triplicate.

The instrumental LOD and LOQ were determined by the standard deviation of the intercept of the calibration curve and were equal to 0.17 and 0.51 mg/L. The respective methods, LOD and LOQ, were found to give values equal to 0.34 and 1.0 mg/kg. These values were 2 or 3 times higher compared to values given by other chromatographic techniques, such as High Performance Liquid chromatography-Fluorescence detection (HPLC-FLD), and they were much higher than that given by Ultra Performance Liquid chromatography-electrospray ionization-tandem mass spectrometry UPLC-ESI/MS^n^ [21,22]. However, the calculated LOD value was considered “fit-for-purpose”, taking into account the reference daily dose of 18 mg/kg as provided in the European Regulation 1169/2011 [3].

**Figure 1:**
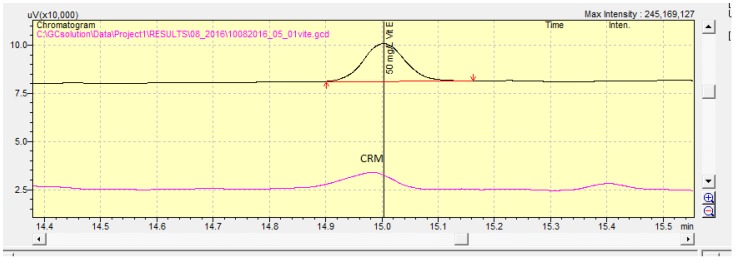
Chromatogram of the certified reference material (CRM; pink line) and a standard solution containing 50 mg/L vitamin E (black line).

Precision experiments were carried out, and the relative standard deviation (%RSD) values achieved from three different concentration levels measured six times under repeatability conditions and six times over two different days under reproducibility conditions were lower than 10% for all the different concentration levels. The repeatability HORRATr and reproducibility HORRAT_R_ values (the observed RSDr divided by the RSDr value estimated from the Horwitz equation using the assumption *r* = 0.66R) achieved from these different concentration levels ranged from 0.18 to 0.25. These values were lower than the crucial value of 2, and the method was fit-for-purpose. For accuracy estimation, the certified reference material FAPAS T10112QC with a certified value of 49.0 ± 12.5 was analyzed six times over two different days by two different analysts (*n* = 12), and the recovery was found to be equal to 99.5 ± 5.9 (Figure 1). The recovery data were within ±25% of the target value, as provided by the certification of the reference material, and for this reason, the method was again considered as fit-for-purpose.

The uncertainty of the method was also calculated on the basis of the Eurachem/Citac Guidelines [18]. The calculated expanded uncertainties were found to be equal to 15.0%, 12.3% and 8.50% of the content of the analyte in milligrams per kilogram for the LOQ, the centroid of the calibration curve, and the upper limit of the linear range, respectively (*k* = 2, Confidence Limit (CL) = 95%). The main sources of the calculated uncertainty were the calibration uncertainty, the bias uncertainty and the precision uncertainty.

### 3.2. Determination of α-Tocopherol Content and Diastase Activity in Real Samples Characterized as Superfoods

The method was performed for the determination of vitamin E in 18 different superfoods produced under ISO 22000 recommendations from Artolife SA, Thiva, Greece. The results proved that the content of α-tocopherol ranged from 4.00 to 88.0 mg/kg. The highest content of vitamin E was found in the sample coded as S13, made from zea wheat (*Triticum dicoccum*) and vanilla. Vanilla contains a high amount of vitamin E, approximately equal to 54.0 mg/kg [23], and *Triticum dicoccum* wheat also contains a high amount of vitamin E (approximately equal to 10 mg/kg) [24,25]. It seemed that the temperature during biscuit making did not lead to a great loss of vitamin E. Similar results of vitamin E loss during bread making were also reported by Leenhardt et al. (2006) [24]. The lowest content of vitamin E was found in the sweet bun made from plums, figs and dates (S4 sample), for which the content was approximately equal to the sum of each ingredient’s content. All superfoods containing nuts and almonds gave a high content of vitamin E, as nuts are commonly known for their vitamin E content. The vitamin E content also depended on the vegetable oils used for the preparation of the bakery products. Sunflower oil was used in the preparation of the sweet buns, breadsticks and biscuits using the same amount in order to obtain comparative results.

With regard to the Regulation (European Union, EU) No. 1169/2011 of the European Parliament and of the Council on the provision of food information to consumers, vitamin E may be declared on the foodstuffs according to their nutrient reference intake [3]. A significant amount of the vitamin can be declared only if a foodstuff contains 15% of the nutrient reference values specified in the current regulation. For vitamin E, the reference intake value is equal to 120 mg/kg, or 15% of the reference intake is equal to 18 mg/kg. According to the Commission Regulation (EU) No. 432/2012, establishing a list of permitted health claims made on foods, other than those referring to the reduction of disease risk and to children’s development and health, the following health claim can be used for a foodstuff containing vitamin E levels higher than the 15% of the reference daily intake: “Vitamin E contributes to the protection of cells from oxidative stress” [26]. Taking into account the serving size of 100 g, the samples coded as S1, S2, S9, S10 and S11 cannot use this specific health claim. In particular, for the sweet bun with plums, nuts, and dates (S12) and biscuits with zea wheat and vanilla, the health claim (S13) can also be used, as well as for the serving size of 35 g (1 biscuit). Summarizing, in the majority of the samples (over 72%), the following health claim can be used on the labeling: “Vitamin E contributes to the protection of cells from oxidative stress”.

## 4. Conclusions

The method presented in this application can be used for the determination of vitamin E in cereal and biscuit products by GC-FID. The GC-FID method proposed is considered fit-for-purpose in terms of precision and accuracy. The results prove that the majority of cereal products are rich in vitamin E. The health claim for vitamin E as specified in Commission Regulation (EU) No. 432/2012 can be used for the majority of the samples. Vanilla and nuts seem to give an excessive amount of vitamin E in bakery products, whereas bakery products containing only fruits seem to give a lower content.

## Figures and Tables

**Table 1:** Mean content of vitamin E in different cereal and bakery products.

Sample Type	Sample Coded As	Ingredients	α-Tocopherol (mg/kg)	% Daily Value
Sweet bun	S1	Oats and honey	15.0	12.5
Sweet bun	S2	Plums, figs and dates	4.00	3.30
Sweet bun	S3	Chocolate, bilberries and raspberries	19.0	15.8
Sweet bun	S4	Nuts, oranges and molasses	34.0	28.3
Sweet bun	S5	Nuts and chocolate	31.2	26.0
Sweet bun	S6	Forest fruits	33.2	27.6
Sweet bun	S7	Whole grain with fruits	34.1	28.4
Sweet bun	S8	Almond	50.2	41.8
Sweet bun	S9	Rye, plums, nuts and raisins	6.12	5.10
Sweet bun	S10	Cereals and nuts	10.0	8.30
Sweet bun	S11	Oats, apple and cinnamon	10.5	8.80
Sweet bun	S12	Plums, nuts and dates	60.0	50.0
Biscuits	S13	*Triticum dicoccum* wheat and vanilla	88.0	73.3
Breadstick	S14	Oats and wheat	18.2	15.2
Breadstick	S15	*Triticum dicoccum* wheat, tomato and oregano	24.2	20.2
Breadstick	S16	*Triticum dicoccum* wheat and sesame	20.0	16.7
Breadstick	S17	*Triticum dicoccum* wheat, whole grain	20.2	16.8
Breadstick	S18	*Triticum dicoccum* wheat and vegetables	20.5	17.1
